# Simulation of a Fully Digital Computing-in-Memory for Non-Volatile Memory for Artificial Intelligence Edge Applications

**DOI:** 10.3390/mi14061175

**Published:** 2023-05-31

**Authors:** Hongyang Hu, Chuancai Feng, Haiyang Zhou, Danian Dong, Xiaoshan Pan, Xiwei Wang, Lu Zhang, Shuaiqi Cheng, Wan Pang, Jing Liu

**Affiliations:** 1 State Key Laboratory of Fabrication Technologies for Integrated Circuits, Institute of Microelectronics of the Chinese Academy of Sciences, Beijing 100029, China; 2 University of Chinese Academy of Sciences, Beijing 101408, China; 3 Institute of Advanced Technology, University of Science and Technology of China, Hefei 230031, China

**Keywords:** computing-in-memory (CIM), NOR-Flash, artificial intelligence, convolutional neural network

## Abstract

In recent years, digital computing in memory (CIM) has been an efficient and high-performance solution in artificial intelligence (AI) edge inference. Nevertheless, digital CIM based on non-volatile memory (NVM) is less discussed for the sophisticated intrinsic physical and electrical behavior of non-volatile devices. In this paper, we propose a fully digital non-volatile CIM (DNV-CIM) macro with compressed coding look-up table (LUT) multiplier (CCLUTM) using the 40 nm technology, which is highly compatible with the standard commodity NOR Flash memory. We also provide a continuous accumulation scheme for machine learning applications. When applied to a modified ResNet18 network trained under the CIFAR-10 dataset, the simulations indicate that the proposed CCLUTM-based DNV-CIM can achieve a peak energy efficiency of 75.18 TOPS/W with 4-bit multiplication and accumulation (MAC) operations.

## 1. Introduction

To break the “memory wall”, computing-in-memory (CIM) has been proposed, and their superiority has been illustrated in AI edge inference. NOR Flash, as a commodity non-volatile memory (NVM) with high parallelism, has been used extensively in CIM. Compared with static random-access memory (SRAM), NOR Flash memory is cheaper with a larger storage capacity. Compared with some emerging non-volatile memristors such as phase change memory (PCM), spin-torque-transfer memory (STT-MRAM), and resistive random access memory (RRAM) [1,2,3], the NOR Flash has stronger stability. Analog CIMs based on NOR Flash have achieved substantial progress for high computing density and energy efficiency, using multi-level cell (MLC) Flash devices. However, the analog CIM is faced with the challenge of accuracy loss.

**Figure 1:**
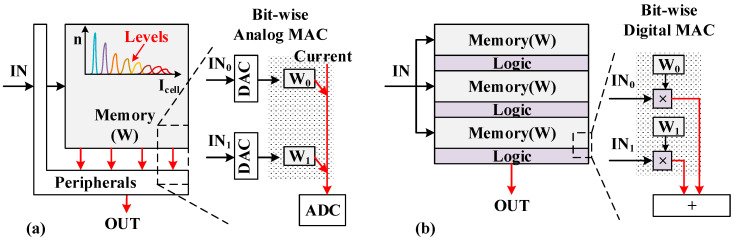
(**a**) Analog CIM structure. (**b**) Digital CIM structure.

As shown in Figure 1a, analog CIM stores the network’s weights in NVM cells and efficiently executes individual MACs in the analog domain, and the full MAC operations can be processed in parallel. For instance, by collecting the current of bit-line (BL), the MAC results of multiple input voltages and weights are obtained by only one measurement. The inherent properties of analog circuits, such as the threshold voltage distribution of Flash cells, the noise, and process fluctuation, can easily lead to accuracy loss. Xiao, T. Patrick, et al. [4] have presented the distribution of MLC cells, that there are crossovers between currents at different levels. It is difficult to ensure the linearity of the BL current. 

In addition, the large-scale integration of analog circuits is hindered due to the high energy and area consumption of an analog-to-digital converter (ADC). There are various approaches to address the limitation of analog computing accuracy in NVM. Xiao, T. Patrick, et al. [4] use a modeled error distribution to approximately represent a 7-bit value, providing an efficiency of 20.1 TOPS/W and 74.3% accuracy for ResNet50. Han, Runze et al. [5] presented a CIM array based on single-level cell (SLC) NOR Flash devices with digital input pulses and successfully handled an application of binarized neural networks (BNN). In these studies, the CIM is executed still in the analog domain and is mainly suitable for specific BNN applications.

Faced with the challenge of accuracy loss of analog CIMs, more recently, digital CIMs are gaining favor for high precision and energy efficiency. As shown in Figure 1b, digital CIM works using digital logic circuits integrated within memory cell arrays. The weights are read out directly and summed by digital adder tree circuits. The operation of multi-bit multiplication and accumulation (MAC) is executed in the digital domain. Nevertheless, the embedded digital circuits in the memory array tend to degrade the storage density. In addition, digital CIM works have been widely discussed in SRAM [6,7,8,9,10]. It is still rarely discussed in NVM, such as NOR Flash memory. 

To address the above challenges, this paper proposes a fully digital NVM CIM (DNV-CIM) macro by using the proposed compressed coding LUT multiplier (CCLUTM) and continuous accumulation scheme. The DNV-CIM takes advantage of high accuracy and high storage density by executing the MAC operation in the digital domain and storing the weights in the NVM. The macro is implemented in 40 nm SLC silicon-oxide-nitride-oxide-silicon (SONOS) technology and can be applied in the deep convolution neural network (CNN), which is a common machine learning architecture. In this paper, the main innovations are as follows. (1) The weight parameters are presented in the form of digital LUT instead of analog conductance, avoiding the influence of process fluctuation on calculation accuracy. (2) The sparsity of weight parameters is taken into consideration by inducing a compressed coding scheme to improve the performance and save memory space further. (3) By inducing a continuous accumulation scheme, the DNV-CIM can continuously process the MAC operations for CNN tasks with low power consumption and with input data multiplexing. (4) The SONOS charge trap memory is well explored as a carrier to CIM for basic properties of low power consumption and simple operational flow. In addition, the circuit structure is fully compatible with commodity Nor Flash products. The proposed digital CIM solution in NVM may play an important role in enabling commercial Flash for highly efficient AI edge inference on CIM.

## 2. Overall Structure of the Proposed DNV-CIM Macro

**Figure 2:**
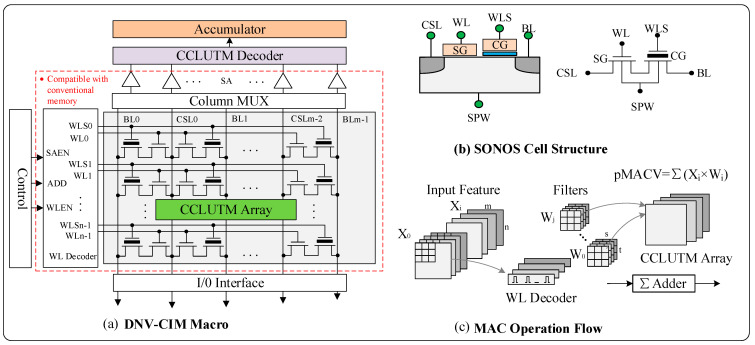
(**a**) The overall structure of the proposed DNV CIM. (**b**) The SONOS structure. (**c**) MAC operation flow.

The overall structure of CCLUTM-based DNV-CIM is shown in Figure 2a. There are some common components with standard commodity NOR Flash memory, such as memory array, word-line (WL) decoder circuit, bit-line (BL) column MUX, and sense amplifier (SA) circuits. The DNV-CIM can be operated in memory and computing modes. In memory mode, the same as conventional memory, data can be erased, programmed, and accessed through the I/O interface. The address signal ADD is passed to the WL decoder and BL MUX module to select specified WL_n-1_ (WLS_n-1_) and BL_m-1_. The SAEN and WLEN, from the top control model, are used as the enable signals for sense amplifiers. The double-tube structure of the SONOS cell structure is shown in Figure 2b. When in computing mode, the DNV-CIM is mainly used to deal with the MAC operations, which constitute more than 90 percent of the computation in the deep CNN [11]. 

Figure 2c shows an example of the operational flow of the 2D CNN computation. It involves inputting matrices Xi (m × n) and filters Wj (i × s × t). The MAC operation is to encode weights data in the form of CCLUM and store them in the memory array. For AI edge inference computation, the input feature data Xi is used as a pointer to CCLUM values by enabling the corresponding WL, then the product results (Xi × Wi) are read out through the SA, decoded by CCLUTM decoder circuit, and summed in the accumulator circuit. In this way, the multiplication operation is simplified to accessing Flash cells in one reading cycle. In addition, the MAC is executed in the digital domain without consideration of the influence of process variations in NVM. 

## 3. CCLUTM

In the conventional LUT-based multiplier, possible product results are stored in LUTs, and the input data works as a pointer to index the specified value. It is expected to store more weight data with less storage space for achieving large storage density. Sparsity is an inherent attribute of the neural network, which can be used to compress the net size and optimize CIM performance. The sparsity of input data is usually used to optimize the CIM circuit. For example, If the input element is 0, the WL decoder skips the data 0 directly to save calculation time and power consumption. Nevertheless, it is rarely discussed for the sparsity of weight in CIM. In this paper, the CCLUTM encoding scheme is proposed to compress LUT size and improve efficiency further. For MAC operation, the product equation is Y = W × A, where A is the input data, and W is the weight parameter. When taking the forward inference operation of a 4-bit quantized neural network as an example, the weights are within the range of [−8:7], and the weights are not modified before mapping into the memory array. All possible values of Y are within the range of {−8 W, 7 W}. Therefore, a memory space of 16 bytes is used to store the product values. In this paper, the input data are compressed from (−8, 7) into (1, 3, 5, 7), and the weights data are also compressed by removing data 0.

**Figure 3:**
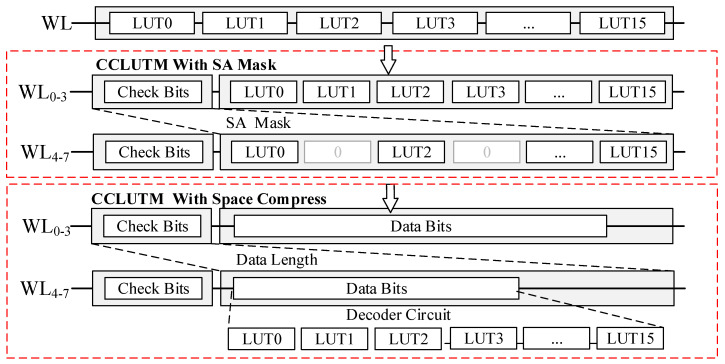
Compression coding scheme.

**Figure 4:**
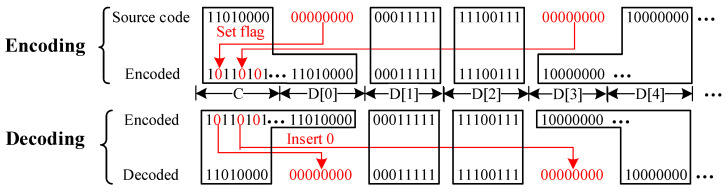
Encoding and decoding principle.

Some studies have found that the weights in CNN often follow the bell-shaped and long-tailed distribution [12]. The weight data 0 occupies most of the percentage. Figure 3 shows the basic compression coding scheme. The 16 LUTs in the same word line (WL) are organized as a group source code. The encoded data is divided into two segments, 16-bit check-bits and a piece of data-bits. Each check-bit presents if the LUT value is an 8-bit 0. Figure 4 shows the principle of encoding and decoding operation. The basic principle of the compression coding scheme is to remove the source code of 8-bit 0 and then set the corresponding check bit to 0. When decoding, the removed data 0 is inserted back. This operation is performed in a pipeline manner over two read cycles, with the first cycle reading flag bits and the second cycle reading corresponding data based on flag bits. The CCLUTM works in two ways: CCLUTM with SA mask and CCLUTM with space compression. In the way of CCLUTM with SA mask, the length of data bits is the same as the original 16 LUTs. When in read mode, the SAs for the LUT with data 0 are masked according to the check bits. In this way, the power consumption is reduced with fewer active SAs, and the operation is easy with an aligned address and simple SA mask logic. In the CCLUTM with space compression, the length of data bits is decoded depending on the values of the check. In this way, the memory space is saved, but the CCLUTM decoder will increase the read latency and power consumption. We optimized the parallel decoding method, as shown in Table 1. 

## 4. Continuous Accumulation Scheme

**Figure 5:**
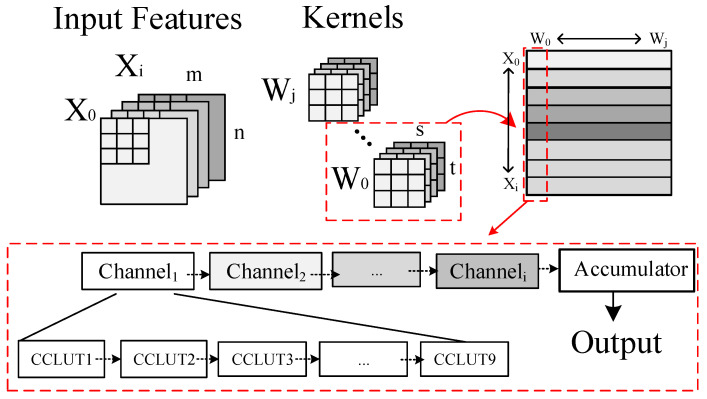
The operational flow of continuous accumulation scheme.

In order to ensure the storage density and successfully realize the CNN application, the continuous accumulation scheme is proposed—as shown in Figure 5. Due to the parallel structure of NOR Flash memory, the kernels with the same input channel are mapped along the same WL. The kernels with different input channels are mapped along the same BL. The continuous accumulation scheme is to accumulate the CCLUT values along the BL for each kernel continuously. 

**Figure 6:**
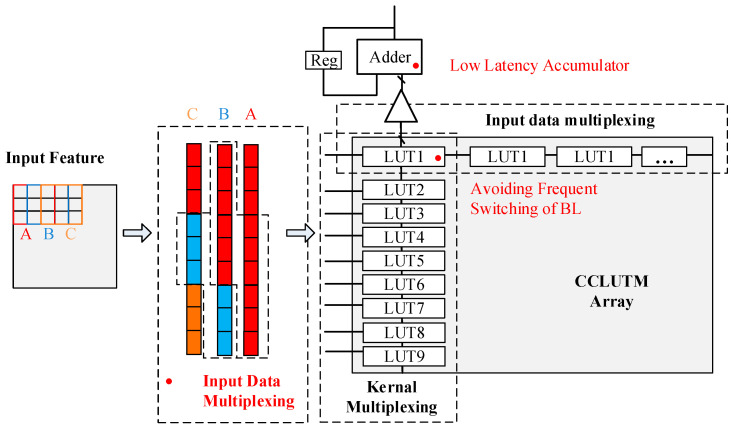
The benefits of continuous accumulation scheme.

The continuous accumulation scheme can bring three benefits as shown in Figure 6. Firstly, the power overhead caused by frequent switching of BL is avoided. Secondly, compared with the adder tree circuit, the accumulator circuit has a lower latency, which will improve the calculation speed. Finally, the input data multiplexing can be used to improve computational efficiency. This scheme can be applied to commodity the Nor Flash memory circuit and also be suitable for the large memory array.

## 5. Experiment

**Figure 7:**
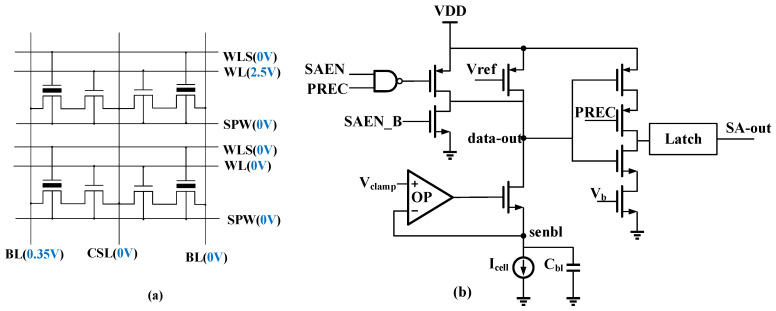
(**a**) The read condition of the SONOS array. (**b**) The SA schematic used in this work.

To evaluate the performance of the proposed solution. The proposed CCLUTM-based DNV-CIM macro is implemented using 40 nm SONOS technology, with a memory size of 1024 × 8192 and 128 SAs. The SONOS charge trap memory is well exploited as a carrier to CIM for intrinsic properties of low power consumption and simple operating flow. For the large on/off ratio of the SONOS cell, cell 0 contributes negligible current. The double-tube structure makes SONOS have better tolerance than other devices, which is necessary for continuous reading. The read condition of the SONOS array is shown in Figure 7a. The voltage of WL is set to 2.5 V, which is equal to V_DDA_ When in read mode. Therefore, there is no on-chip pump circuit needed for a read operation, which is an energy-hungry module in conventional Nor Flash memory. The BL is clamped to 0.35 V, and other signals are set to 0 V. Figure 7b shows a SA circuit used in conventional Nor Flash memory, which is also used in our DNV-CIM macro. It consists of a clamping circuit, an inverter, and an output latch.

## 6. Results

**Figure 8:**
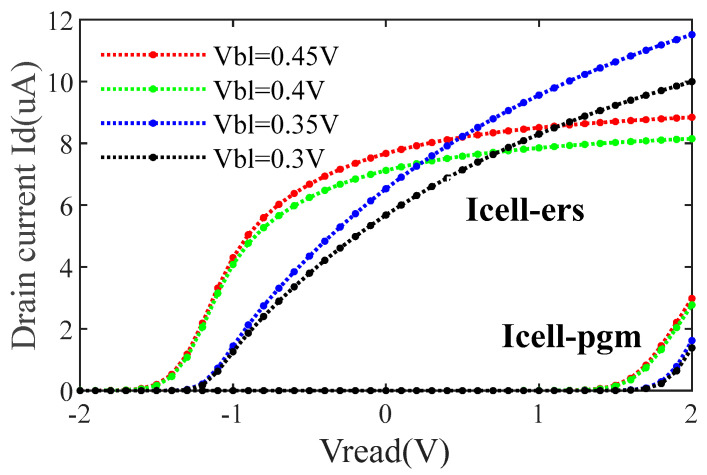
I-V characteristic curves of SONOS Cell.

**Figure 9:**
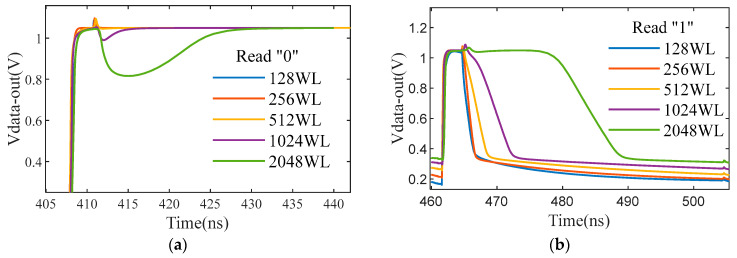
(**a**) The simulation of SA operation time under different WL lengths for reading “0”. (**b**) The simulation of SA operation time under different WL lengths for reading “1”.

Figure 8 shows the I–V characteristics of the SONOS cell under different BL biases. As can be seen from the figure, when Vread = 0 V, the erased cell current Icell_ers increases with the increase of clamping voltage Vbl. The lower clamping voltage will improve the reading speed of SA and reduce power consumption but will affect the accuracy of the reading. When setting Vbl = 0.35 V, the window between Icell_ers and Icell_pgm approximately equals 6 μA. The performance of SA is evaluated at loads of different WL lengths. As shown in Figure 9, when the WL length is 128, the SA behaves at the fastest speed of 6 ns read time and lowest power consumption of 8.3 μA. When the WL length is 2048, the SA behaves at the slowest speed of 26 ns read time and highest power consumption of 42.5 μA. The detailed simulation results are shown in Table 2. The power of DNV-CIM is computed based on SPICE simulations. To compromise performance and storage density, the DNV-CIM is designed with a WL length of 1024, which corresponds with the parasitic capacitance of 1024 fF.

**Figure 10:**
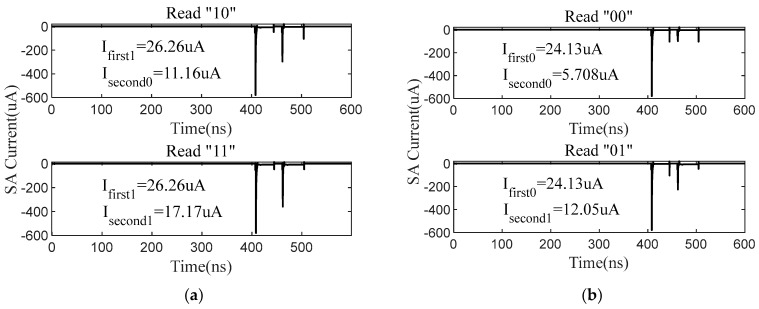
(**a**) The simulation of SA current in continuous read mode for read data “10”, “11”. (**b**) The simulation of SA current in continuous read mode for read data “00”, “01”.

**Figure 11:**
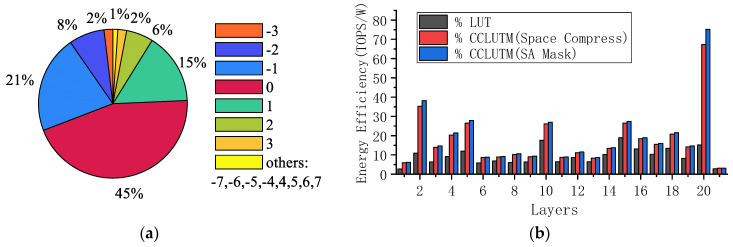
(**a**) Weight sparsity of Resnet18 network. (**b**) The energy efficiency of DNV-CIM.

The SA is set to work in continuous read mode. The first cycle current represents the maximum result of SA operations, and then the second cycle current represents the current in continuous read mode. As shown in Figure 10, for the second read cycle, the minimum current of SA is 5.708 μA in the case of read data “00”. In addition, the maximum current of SA is 17.17 μA in the case of read data ”11”. Compared with the current in the first cycle, the current of SA in continuous mode is significantly reduced. The power of the accumulator and CCLUTM decoder circuit are 63.55 μW and 30.37 μW based on SPICE simulations. The test of the proposed CCLUTM-based DNV-CIM is executed on a modified ResNet18 model under CIFAR-10, which comprises 20 convolution layers and one fully connected layer. The sparsity of weights is obvious, as shown in Figure 11a. The weights of 0 occupy 44.85 percent. Figure 11b shows the energy efficiency. With the use of the proposed CCLUTM, the proposed DNV-CIM can achieve 93.04% inference accuracy with 4-bit MAC operations. When working in the way of an SA mask, the DNV-CIM can achieve a peak energy efficiency of 75.18 TOPS/W. However, when working in the way of space compression, the DNV-CIM can achieve a peak energy efficiency of 67.25 TOPS/W with a total space compression rate of 51%.

Table 3 presents the energy efficiency and inference accuracy of DNV-CIM compared with existing CIM works. Compared with SRAM-CIM work [13], larger memory density can be achieved. Therefore, it can be applied to more general neural network applications that use large NVMs without weight data reloading. Compared with previous CIM work based on SLC NOR-Flash [5], which is limited to binary neural networks (BNN), more common neural networks can be supported. Compared with analog CIM arts [4,14,15], the proposed DNV-CIM achieved higher inference accuracy and with >2× energy efficiency. The benefits mainly arise from the proposed CCLUTM and continuous accumulation scheme.

## 7. Conclusions

An 8 Mb fully digital NVM CIM structure was implemented and analyzed with the 40 nm technology node. The CCLUTM and continuous accumulation scheme were proposed to realize high energy efficiency and save memory space. The weight sparsity is taken into consideration. When applied to a modified ResNet18 Network, the proposed CCLUTM-based DNV-CIM can achieve an inference accuracy of 93.04% under CIFAR-10. This CIM structure also achieved a peak energy efficiency of 75.18 TOPS/W in the way of an SA mask and a 67.25 TOPS/W with 51% memory space compression for 4-bit MAC operations. The CCLUTM is well-matched to the properties of modern CNN networks, whose weights are heavily skewed toward zero. These results indicate that the DNV-CIM can be used for a successful demonstration of the high performance of digital CIM in NVM.

## Figures and Tables

**Table 1:** Decoding method of proposed compression coding scheme.

Decoding Method	
$\mathrm{Data}[0]=\left\{\begin{array}{c} 8\prime d0\\ D\;[0] \end{array}\right.$	C [0] = 0
	C [0] = 1
$\mathrm{Data}[1]=\left\{\begin{array}{c} 8\prime d0\\ D[C[0]] \end{array}\right.$	C [1] = 0
	C [1] = 1
$\mathrm{Data}[n]=\left\{\begin{array}{c} 8\prime d0\\ D\;[\sum_{i=0}^{n-1}C[i]] \end{array}\right.$	C [n] = 0
	C [n] = 1

**Table 2:** SA comprehensive simulation results.

WL Length	128	256	512	1024	2048
Time/ns	6	6.4	7.9	10.9	26
Current/μA	8.3	10.9	15.3	25.2	42.5

Note: The current represents the average result of SA sampling current of data “1” and “0”.

**Table 3:** Overall performance evaluation.

	DAC [13]	TCAS-I [5]	TCAS-I [4]	ISCAS [15]	ISCAS [14]	This Work
Technology Node	65 nm	-	40 nm	65 nm	40 nm	40 nm
Memory Capacity	4 Kb	64 Kb	512 kb	1 Mb	1 Mb	8 Mb
Accelerate Type	Digital SRAM	Analog (SLC NOR Flash)	Analog (MLC NOR Flash)	Analog (MLC NOR Flash)	Analog (MLC NOR Flash)	Digital (SLC NOR Flash)
Data Resolution	1 bit	1 bit	8 bit	4 bit	4 bit	4 bit
Energy Efficiency (TOPS/W)	>50 (1 b/1 b) (BNN)	- (BNN)	20.1 (4 b/4 b) (ResNet50)	- BSIM3v3	35.6 (4 b/4 b) (peak) (VGG-16)	75.18 (4 b/4 b) 67.25 (4 b/4 b) (peak) (ResNet18)
Accuracy	87.46%	-	74.3% (loss 2.16%)	97.1% (loss 0.7%)	92.73%	93.04%

Note: The performance of TCAS-I [9], TCAS-I [13], ISCAS [14], ISCAS [15], and this work are simulated. The performance of DAC [13] is evaluated by a tap-out chip based on SRAM. The Energy Efficiency values of 75.18 and 67.25 were evaluated when CCLUTM works in different ways: CCLUTM with SA mask and CCLUTM with space compression.
